# Caring for adolescents and young adults (living with HIV) and the disclosure of a stigma

**DOI:** 10.3389/fpubh.2023.1150769

**Published:** 2023-03-23

**Authors:** Maria Leticia Santos Cruz, Mariana Queiroz Darmont, Simone Souza Monteiro

**Affiliations:** ^1^Infectious Diseases Department, Hospital Federal dos Servidores do Estado, Rio de Janeiro, Brazil; ^2^Fernandes Figueira Institute (IFF), Oswaldo Cruz Foundation, Rio de Janeiro, Brazil; ^3^Oswaldo Cruz Institute, Oswaldo Cruz Foundation (Fiocruz), Rio de Janeiro, Paraná, Brazil

**Keywords:** HIV-stigma, disclosure, socialization, vertical transmission, group activities

## Introduction

Stigma is often defined as an attribute that discloses an undesirable condition. The nature of this condition may be biological or moral and causes differentiation and devaluation of stigmatized persons when compared to everyone else who is considered normal. In modern societies there are many conditions that may become a stigma, such as race/ethnicity, poverty, unemployment, low educational attainment, social class, sexual orientation, drug use, disabilities, or some diseases, such as AIDS. It is therefore appropriate to reflect on how health and wellbeing are affected by the convergence of multiple stigmatized identities through the analysis of the interaction of systems of oppression or discrimination—related to social inequalities, gender, color/race, sexual orientation, among others—at the social, community and individual level (1). In light of this, analysis of AIDS-related stigma needs to consider its interactions with other processes of stigmatization that generate hierarchy and social categorization. A Brazilian study from 2015 found association between factors such as sex work, lower education and drug use and HIV stigma (2).

Although HIV epidemics in Brazil affects more strongly groups of men who have sex with men and sex workers, many children, adolescents and their parents are living with HIV (3). HIV-related stigma in Brazil and other parts of the world, is a cause of suffering and a barrier to linkage to care and treatment adherence.

In 2015, our group published a study based on Goffman's theory about the “moral career” of stigmatized individuals (4). Therein, we analyzed the primary socialization of children living with HIV as a process that may contribute to the formation of stigmatized individuals. We found that the disclosure of diagnosis played a key role in that setting. In the present study, we propose to review Goffman's theory informed by our experiences caring for adolescents and young people living with HIV (YPHIV) in Rio de Janeiro, Brazil.

## Disclosure vs. non-disclosure: Respecting boundaries

Antiretrovirals have made it possible for vertically infected children to survive into adolescence requiring families, health teams and stakeholders to develop care strategies, including techniques for the disclosure of diagnosis. Such strategies should be elaborated within the social and cultural context, considering family relationships, meanings, and representations.

Currently, diagnosis disclosure has become a routine practice in caring for YPHIV. When, to whom, with whom, what and how to disclose are important aspects of this process. How should a potential stigma be disclosed without causing damage and suffering to the individual in question? Fear of stigma causes difficulties to disclosure in many families (5).

Goffman (6) analyzes how the stigmatized person learns about their condition and how they cope and react to the stigmatization within social interactions. George (7) points out that the opposite of disclosure is concealment and that everyone has something that they do not wish to reveal, and these secrets pervade social relationships. A secret introduces differences and, as a privilege, is a source of power. Foucault (8), discussing the concept of biopower, points that once revealed, secrets contribute to hierarchical relationships that perpetuate violence and oppression. Therefore, since diagnosis disclosure is part of routine care activities for families and health care workers, we must determine how to avoid causing or perpetuating violence and oppression in different scenarios of disclosure.

Current literature relating stigma and HIV disclosure is focused on experiences with adult populations (9–12). A Brazilian study on disclosure to children brought the interesting aspect of silence as a type of communication “*among people who need to live in silence*” due to the stigma and discrimination internalized by these families (13).

Living with HIV has historically been associated with practices perceived as deviant, like drug use and sex work. In the context of socially-situated bodies, HIV diagnosis becomes an agent and object of hierarchy and social categorization. Caregivers must be familiar with the social context and values of families l. AIDS-related stigma is superimposed upon other stigmas that generate social vulnerability, such as race/ethnicity, disabilities, social class, income, country/territory of origin, gender identity, and sexual orientation (2). These overlapping stigmas affect subjective personal experiences, an individual's personal and professional development, and perceptions about heritage, youth, older generations, kinship, care and social support.

Due to these stigmas, caring for YPHIV involves not only the practice of medicine, but a broader social context. In this setting, some families may choose not to disclose a family member's HIV status to protect the individual from stigmatization.

## Goffman's theory in practice

To contextualize the present discussion, we will briefly review Goffman's theory about how individuals “learn” that they possess an attribute that may cause social discrimination and adjust their behavior accordingly. The whole process begins with individuals internalizing the belief of the hegemonic social group, considered normal people, about what a particular stigma means. What happens next, when someone becomes aware that he/she has an attribute that is seen as negative?

The first process or practice that Goffman analyzes is the socialization of individuals who have a congenital condition that is considered negative. This process typically occurs in families in which a child living with HIV becomes ill due to immunodeficiency. Misinformation about the disease and how treatment can alter the natural history of infection impact the socialization of these children early in life. In some cases, those who raise these children may view them as condemned.

The second practice investigated by Goffman is keeping the diagnosis a secret, including to the child itself. This practice involves the family's attempt to create a protective social barrier to avoid disclosing the child's true condition. It is rarely possible to keep this secret for the child's entire life. In these situations, the truth comes out either naturally or by accident and may cause confusion, uncertainty, fear, or other harmful emotions.

The third process or practice has to do with stigmatization that takes place or comes to light later in life. We have cared for children that were infected with HIV perinatally but experienced slow progression of disease such that their HIV status was only disclosed to them at 10 years of age or older. It is crucial to provide these individuals with clear information and support that helps them cope with their new identity.

The fourth set of processes or practices involves children raised outside of nuclear families, such as those in foster care. Children raised in foster care facilities come to view their HIV diagnosis, treatment, and other related issues as part of their normal routines. For these individuals, conflicts may arise at the conclusion of foster care (usually 16–18 years of age) when they have to adapt to different living arrangements.

When an individual's HIV status is kept secret, anyone who knows the secret can use it to threaten to the individual in question. YPHIV may also have other attributes that they wish to keep secret such as their sexual orientation, whether they are orphans, disabilities, and country/territory of origin. Like HIV status, these other secrets could also potentially be used for blackmail or coercion.

## Learning from trajectories after disclosure

In 2021, we published an article about youth in transition to an adult outpatient HIV clinic (14). We aimed to investigate experiences of HIV stigma and its repercussions on their quality of life. We have studied these individuals' development after the disclosure of their HIV diagnosis, keeping track of what information was shared with others and what was kept secret. There were instances in which these youth reported that they were discriminated against or treated abusively by healthcare workers (HCW). It is of utmost importance that this should be avoided. HCW should keep in mind that the disclosure of HIV status is a component of medical care and is regulated by medical and ethical guidelines. HCW should also be sensitive to the fact that the disclosure of HIV diagnosis has complex social implications. It is hoped that by adopting this perspective, HCW can avoid contributing to social processes that coerce or oppress YPHIV. Moreover, we have observed that the practice of disclosing HIV diagnosis encompasses not only the medical sphere but also the social sphere. In this setting, HCW becomes involved in the sphere of the patient's familial and social relationships. The role that HCW plays in this sphere is particularly important as he or she has access to confidential information that is potentially stigmatizing. Our findings tend to support the hypothesis that concerns about disclosure may reduce adherence to antiretrovirals and impede the successful transition to the adult clinic. In the population that we follow, individuals tend to keep their HIV status and other stigmatized attributes secret for an extended period of time.

## Group activities

Group experience in the context of HIV/AIDS, presents diversified approaches: support groups, open, closed, transitory, permanent, psychoeducational and mutual help groups (15). The group becomes a real and symbolic place for sociability and coping with the pervasive discrimination caused by the stigmas that afflict the lives of those living with HIV, where belonging and solidarity are integrated into perceptions and practices of care and mutual aid.

The group of YPHIV, whether within health practices, activism, or in digital spaces, illustrate sociability brought together by the feeling of belonging. As a practice, it presents a powerful resource for establishing friendship bonds, sharing stories and experiences, building autonomy in decision-making regarding treatment, strengthening bonds with the team, and facing fear of stigma. It also fosters solidarity, which is an important element of inclusion and belonging.

However, there is a fine line between inclusion and segregation, present in the complexity of living with a stigmatizing disease. Categorizations with reproduction of moral values, can reinforce prejudices and generate practices of violence (16, 17). For example, when forming a group of YPHIV, one cannot fall into the dualistic fallacy that categorizes vertically infected as helpless victims vs. sexually infected as responsible and guilty. On the other hand, in the heterogeneous group, YPHIV are considered to have dangerous bodies (17) that must have their desires protected, to the detriment of those free of HIV, marking the difference between bodies, reverberating beyond moral and health issues, to other legal conflicts such as criminalization.

Groups restricted to those living with HIV, can reinforce a fragmented model of society, losing the opportunity to work on inclusion, prevention, health care in its diversities, erroneously reinforcing the idea that AIDS is a problem for just one group, echoing a segregationist stance. On the other hand, groups formed only by PLHIV are an important tool to build a support network, sharing practices and experiences based on a new identity that includes the diagnosis and its social representations which include responses to face stigma. We worked with groups of YPHIV, aware of diagnosis, regardless of how they acquired it, mixing an educational and supportive character based on mutual help and encouraging participation of young seronegative partners, siblings or other related youths.

Based on our clinical experiences and literature data (18), we believe that groups of YPHIV are more effective at confronting stigma. Data also show that publicizing the diagnosis is important to face the stigma (19), so that living with HIV/AIDS cease to be an individual problem and becomes outlined in a relational and political perspective (20).

## Conclusion

Inspired by Goffman's theory, analyzing processes and practices of socialization of children and young people living with HIV/AIDS, gives us the opportunity to understand how these individuals are situated with networks of social relationships. When an individual learns that he or she bears a stigma, they also become a member of the community of stigmatized people and have the opportunity to interact with other people who share the same disadvantageous condition.

The testimonies of YPHIV highlight the importance of new knowledge and discussions about this process of becoming an adult with a long-term illness, its health effects, the apprehensions and fears that this generates, challenges and achievements they have attained. We hope that in a near future the challenge of living with HIV will cease to be the problem of one particular community and become an issue that society as a whole can collaborate to address.

## Author contributions

MC mentored the article and wrote the first draft. After authors input she wrote the final version of the manuscript. MD participated in conceptualization and writing the manuscript. SM participated writing the manuscript. All authors contributed to the article and approved the submitted version.
